# Cancer treatments transform residual cancer cell phenotype

**DOI:** 10.1186/1475-2867-11-1

**Published:** 2011-01-07

**Authors:** William W Harless

**Affiliations:** 1 Department of Medical Oncology, Waikato District Health Board, Hamilton, New Zealand

## Abstract

**Background:**

Physiologic wound repair and tissue regeneration are associated with distinct cellular behaviors triggered by tissue damage. Normally quiescent stem cells proliferate to regenerate damaged tissue, while relatively immobile epithelial cells can transform into a motile, tissue invasive phenotype through a partial epithelial-mesenchymal transition. These distinct cellular behaviors may have particular relevance to how cancer cells can be predicted to behave after treatments damaging a tumor.

**Presentation of the hypothesis:**

Surgery, chemotherapy, and radiation therapy trigger highly conserved wound healing pathways that: (1) facilitate the phenotypic transformation of surviving cancer cells into a highly mobile, metastatic phenotype through an EMT or epithelial-mesenchymal transition and (2) induce residual cancer stem cell proliferation.

**Testing the hypothesis:**

Tissue damage caused by cancer treatments will trigger the release of distinct cytokines with established roles in physiologic wound healing, EMT induction, and stem cell activation. They will be released rapidly after treatment and detectable in the patient's blood. Careful histologic evaluation of cancerous tissue before and after treatment will reveal cellular changes suggestive of EMT induction (down regulation of cytokeratin expression) and cancer stem cell enrichment (stem cell markers upregulated).

**Implications of the hypothesis:**

Cancer cells surviving treatment will be more capable of metastasis and resistant to conventional therapies than the pre-treatment population of cancer cells. These changes will develop rapidly after treatment and, in distinct contrast to selection pressures fostering such changes, be triggered by highly conserved wound repair signals released after tissue damage. This pattern of tissue (tumor) repair may be amenable to treatment intervention at the time it is upregulated.

## Background

Cancers of epithelial origin account for 90% of cancer deaths worldwide. Their behavior has been compared to an uncontrolled wound healing process for over a century [1]. In more recent times Dvorak developed this comparison further, describing the differences and similarites between physiologic wound healing and the cancer induced formation of tumor stroma [2].

A distinct cellular behavior observed during wound repair and tissue regeneration is epithelial mesenchymal transition or EMT. An EMT is not a single phenotype, but a general description of a cellular plasticity that can vary from a transient increase in cellular mobility to a complete molecular reprogramming [3]. It occurs in response to different physiologic challenges, including during embryonic development, tissue regeneration, and cancer progression [4]. An EMT can be understood as a biologic process enabling an epithelial cell to assume a mesenchymal-like phenotype, providing that cell with distinct capabilities. These include an enhanced ability to migrate effectively; invade and degrade tissue through matrix metalloproteinase (MMP) expression; an ability to synthesize extracellular matrix components; and a resistance to apoptosis during the anchorage independent conditions associated with migration.

**Figure 1:**
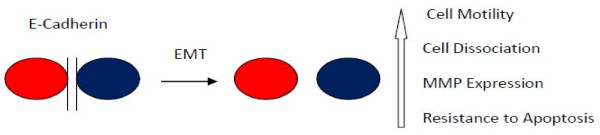
**Epithelial-Mesenchymal Transition**. Normally epithelial cells are held together tightly at cell-cell adherens junctions via glycoprotein E-cadherin. An EMT leads to the breakdown of these junctions and a motile (mesenchymal) and tissue invasive phenotype.

Fundamentally, EMT is a cellular alteration permitting enhanced migration (Figure 1). Normally, epithelial cells are held together tightly at junctions containing E-cadherin in complexes with catenins linked to the actin cytoskeleton, limiting their migration. The dissolution of these adhesive E-cadherin junctions is the hallmark of EMT. This can occur through the down regulation of E-cadherin expression via negative transcriptional activators such as Snail and Twist [5,6], as well as through a growth factor induced relocalization of E-cadherin [7,8].

A good example of an EMT like process occurring during wound healing is re-epithelialization. After incisional injury keratinocytes undergo transient phenotypic changes similar to the more complete EMT changes noted during developmental processes such as gastrulation [9]. These changes include an enhanced migratory ability through the disruption of cadherens junctions and the ability to degrade tissue through metalloproteinase expression. Another good example of an EMT like process occurring during wound healing is the transformation of ovarian epithelial cells to a mesenchymal phenotype under the influence of EGF during the post-ovulatory repair of the damaged surface epithelium [10].

The molecular signals inducing EMT during wound repair appear to emanate from damaged tissue microenvironments. Tissue injury triggers an acute inflammatory response resulting in activation of the coagulation cascade; platelet aggregation; and the proliferation of activated fibroblasts/inflammatory cells at the injury site [11]. This induces the release of numerous soluble inflammatory molecules and growth factors [12]. TGF beta signaling, in cooperation with activation of various receptor tyrosine kinases, is particularly important to the induction of EMT in both physiologic and pathologic settings [13]. The growth factors recognizing these receptors with the strongest links to EMT induction include EGF [10], FGF [14], HGF [15], PDGF [16], and IGF [17].

Closely aligned with wound healing is tissue regeneration. Tissue specific stem cells are responsible for tissue maintenance during the life of the organism, and they can self-renew and produce daughter cells that can differentiate into the more specialized cells comprising the bulk of the tissue [18]. The molecular signals inducing stem cell activation/proliferation are only now being discovered, but similar to EMT induction, tissue damage appears to be an important trigger. In the adult Drosophila intestine, which is an excellent model to study stem cell behavior because intestinal stem cells are the only mitotically active cell, tissue damage triggers a rapid intestinal stem cell activation and proliferation [19]. The mouse intestine is more complicated. The three specialized cell types that comprise colonic epithelium (enterocytes, goblet, and enteroendocrine cells) are replenished by a population of colonic stem cells located in distinct niches in the subepithelium known as the crypts of Lieberkuhn. Tissue injury induces proliferation and expansion of this cell population represented by an elongation of these crypts [20]. Similarly, in the lungs of mice suffering inhalational injury, the rapid generation of large clonal cell patches representing active stem cell proliferation is observed [21].

## Presentation of the hypothesis

**Figure 2:**
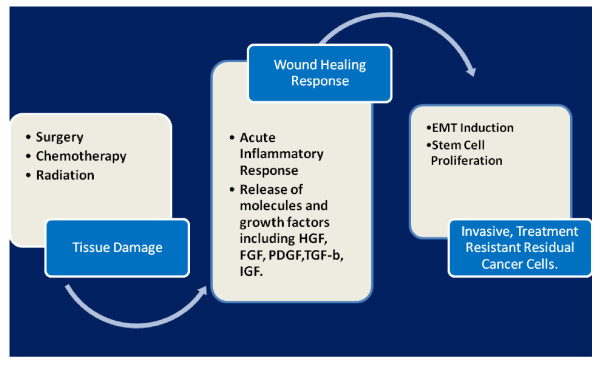
**Hypothesis Schema**. Treatments that damage a cancerous epithelial tumor are predicted to trigger a wound healing response that will lead to EMT induction and cancer stem cell activation and proliferation as an adaptive response to tissue injury. This response, in turn, is predicted to render surviving cancer cells resistant to conventional therapies and more capable of metastatic spread.

Surgery, chemotherapy, and radiation therapy, by damaging both normal tissue as well as cancerous tissue, triggers the release of highly conserved tissue repair molecules that can facilitate both EMT induction and stem cell activation in surviving cancer cells. These molecules will be released rapidly after any treatment that damages the tumor, paralleling the temporal pattern of their rapid release during the acute inflammatory phase of physiologic wound repair [11]. (Figure 2).

## Testing the Hypothesis

That selection pressures imposed by cancer treatments can lead to phenotypic changes in surviving cancer cells is known. For example, a subclone of cancer cells resistant to a given drug will be selected by that treatment [22]. But what is not known is that damage to a tumor through cancer treatments may also induce phenotypic changes in surviving cancer cells purely as a highly choreographed response to tissue injury, and that such changes can facilitate the rapid emergence of a highly aggressive, treatment resistant cancer cell phenotype.

To test this hypothesis acute inflammatory mediators and cytokine levels important in physiologic wound repair, EMT induction, and stem cell activation should be evaluated immediately before and after initial treatment against cancer. Post-treatment sampling should be taken at 24, 48, 72, and 96 hours after treatment as well as one, two weeks, and one month after treatment. These time points encompass the time frame associated with the acute and proliferative phases of a physiologic wound repair response [11,12].

A distinct and replicable "wound healing pattern" should be detectable in the serum or plasma of patients after any cancer treatment, including surgery, chemotherapy, or radiation. This pattern will reveal the upregulated expression of growth factors with established roles in EMT induction such as TGF-beta, HGF, EGF, FGF, PDGF, and IGF as well as molecules now known or eventually discovered to be important in stem cell activation. Because cancer treatments will damage normal tissue as well as cancerous tissue, an important corollary experiment is to sample molecular expression profiles during the perioperative period in both benign and neoplastic surgeries to see what may be unique about cancer surgery. These surgeries should parallel each other as much as possible in terms of the amount of tissue damage to help limit the noise of a systemic wound repair response to clarify what signals may be unique about neoplastic surgery. For example, compare the acute inflammatory expression profile in patients who undergo wide local excision of a breast carcinoma with patients who have surgical removal of benign tumors such as fibroadenomas.

Histologic comparison of cancerous tissue before and after treatment for evidence of EMT induction and stem cell enrichment is another important experiment that should be done. There is already evidence that cancer treatments can result in residual cancer stem cell enrichment, as conventional treatments can select for treatment resistant cancer stem cells [22,23].

Distinguishing between residual cancer stem cell enrichment triggered by a selection process or through the release of tissue repair growth factors may be difficult, particularly after treatment with chemotherapy or radiation therapy. To overcome this limitation one could also compare tumor tissue before and after biopsy. A core biopsy may damage tissue enough to induce a wound repair response capable of inducing EMT and stem cell activation. Careful comparison of core tissue biopsy specimens with subsequent surgically removed tumor tissue would be predicted to reveal the upregulated expression of both stem cell markers as well as markers of EMT. Such changes, if observed, could not be considered as resulting from a selection process.

## Implications of the Hypothesis

The remarkable phenotypic plasticity of epithelial cells is integral to an adaptive repertoire necessary to form tissue during development, as well as to regenerate and repair damaged tissue during the life of the organism. EMT induction confers a migratory and invasive epithelial phenotype critical to tissue repair; stem cell activation provides the epithelial cell precursors necessary to regenerate tissue. If this phenotypic plasticity and the molecular triggers of that plasticity are preserved in malignancies of epithelial origin, a profound shift needs to occur in how we treat these tumors.

A rapid, even transient, phenotypic shift in cancer cells to a more migratory, metastatic phenotype in response to a treatment induced release of tissue repair signals has significant clinical implications. Cancer cell populations that have undergone EMT or that are enriched for cancer stem cells are highly resistant to ionizing radiation, conventional chemotherapy, and highly tumorigenic [24]. Disrupting the highly conserved cytokine/signaling pathways important in EMT induction and stem cell activation at the time they are released could prove highly effective as a treatment strategy against residual cancer cells, preventing the emergence of a treatment resistant, metastatic cancer cell phenotype. Although this clinical strategy could be employed after any treatment damaging the tumor, it may be particularly effective in the potentially curative adjuvant setting after the primary tumor has been surgically removed, limiting the ability of any surviving cancer cell population to proliferate and metastasize.

Merging the ancient concept that cancer behaves like an aberrant wound healing process with our emerging understanding of the importance of cancer stem cells in tumor survival and regeneration provides a powerful conceptual insight. This insight can be used to predict how cancers of epithelial origin will behave in response to cancer treatments. This knowledge, in turn, should guide future treatment strategies and clinical trials. These strategies should include not only what treatments are likely to be effective but when those treatments are likely to be effective, given that the treatments themselves will induce a highly conserved tissue repair response that can fundamentally alter the phenotype of the cancer cells that survive treatment.

## List of Abbreviations

EGF: epidermal growth factor; FGF: fibroblast growth factor; HGF: hepatocyte growth factor; IGF: insulin growth factor; PDGF: platelet derived growth factor; TGF-b: transforming growth factor beta.

## Competing interests

The authors declare that they have no competing interests.
